# Counter-verification in performance-based financing: key insights from the Côte d’Ivoire experience

**DOI:** 10.1080/16549716.2025.2483072

**Published:** 2025-12-18

**Authors:** Joël Arthur Kiendrébéogo, Kouadjo San Boris Bediakon, Jean-Pierre Tsafack, Marin Passerat De Silans, Robert Yao Kouakou, Clovis Kouassi Konan, Sosthène Dougrou, Xavier Lannuzel, Serge Mayaka Manitu

**Affiliations:** a Department of Public Health, University Joseph Ki-Zerbo, Ouagadougou, Burkina Faso; b Department of Research, Expertise and Capacity Building, Recherche Pour la Santé et Le Développement (RESADE), Ouagadougou, Burkina Faso; c Heidelberg Institute of Global Health, Medical Faculty and University Hospital, Heidelberg University, Heidelberg, Germany; d Institute of Tropical Medicine, Department of Public Health, Antwerp, Belgium; e Université Jean Lorougnon Guédé, Training and Research Unit in Economics and Management Sciences, Daloa, Côte d’Ivoire; f Health Department, Ginger INTERNATIONAL/Independent External Verification Agency, Abidjan, Côte d’Ivoire; g Health Division, Ginger INTERNATIONAL, Issy-les-Moulineaux, Île-de-France, France; h Ministry of Health, Public Hygiene and Universal Health Coverage, World Bank Health Projects Coordination Unit, Abidjan, Côte d’Ivoire; i Ministry of Health, Public Hygiene and Universal Health Coverage, Technical Secretariat for Strategic Health Purchasing, Abidjan, Côte d’Ivoire; j Ecole de Santé Publique de Kinshasa, Université de Kinshasa, Kinshasa, RDC

**Keywords:** Performance-based financing, verification, counter-verification, pay-for-performance, Côte d’ivoire

## Abstract

**Background:**

Performance-based financing (PBF) is a key health financing reform in many sub-Saharan African countries, with verification being a critical component. Counter-verification, designed to enhance the credibility of verification, remains largely under-documented and under-researched. This study examines the counter-verification activities within Côte d’Ivoire’s PBF program, providing insights into its implementation and challenges.

**Methods:**

We performed a content analysis of 14 quarterly counter-verification reports spanning from Q4 2016 to Q3 2023, supplemented by brief, informal discussions. Using the READ approach proposed by Dalglish et al., we systematically prepared the materials, extracted and analyzed data, and distilled the findings. Our analysis focused on discrepancies between verified and counter-verified data, identifying and detailing the underlying causes of these inconsistencies.

**Results:**

Our analysis revealed mixed results when comparing the verified and counter-verified data. While discrepancies decreased over time for some activities, others remained consistent or even worsened. The primary causes of discrepancies included inadequate record-keeping and failure to meet indicator validation criteria. Challenges in retrieving documents for counter-verification were linked to poorly managed physical and electronic archiving systems, as well as a lack of qualified personnel. Furthermore, the audit of administrative and financial procedures, especially regarding invoice issuance and the use of PBF funds, highlighted several shortcomings.

**Conclusions:**

Counter-verification is a critical component of PBF programs, designed to strengthen confidence in verifications, correct errors or weaknesses through sanctions or recommendations, and prevent misconduct by both providers and verifiers. To optimize its impact, counter-verification must be systematically integrated into the design of PBF programs.

## PAPER CONTEXT

- **Main findings:** The comparative analysis of verified and counter-verified data revealed inconsistent outcomes. Key issues included inadequate record-keeping, poorly managed archiving systems, and a lack of qualified staff. Additionally, audits of administrative and financial procedures, particularly regarding invoicing and the use of performance-based financing funds, uncovered significant deficiencies.
- **Added knowledge:** While counter-verification is incorporated into some performance-based financing programs, its implementation remains under-documented. This article seeks to address this gap by examining the role of counter-verification in performance-based financing programs and its broader contribution to strategic health purchasing mechanisms.
- **Global health impact for policy and action:** Counter-verification can enhance the effectiveness of pay-for-performance policies and similar programs. To maximize its value, it should be strategically designed to minimize costs while maintaining its deterrent effect and ensuring that its findings are effectively applied.

## Background

Performance-based financing (PBF) has attracted significant attention in many sub-Saharan African countries over the past two decades. Despite ongoing debates about its value for money and its effectiveness in improving health system performance and health indicators [1–3], PBF is being implemented to varying extents, ranging from pilot projects to national policies [4]. Some scholars caution against focusing too narrowly on the details of PBF, urging instead an emphasis on its broader potential to drive systemic health reforms [5–7]. While challenges in its implementation may lead to mixed results [8–10], PBF holds promise as a pathway to strategic health purchasing [11]. This approach involves continuously seeking the most effective interventions, identifying the best providers, and optimizing payment mechanisms and contracting arrangements [12]. PBF has introduced new methodologies and highlighted key features essential for improving health systems, including output-based payments, direct fund transfers to service providers, provider autonomy, enforceable contracts, and the separation of functions such as regulation, service delivery, purchasing, payment, and verification [6,13,14].

Verification is a fundamental component of any PBF program. When conducted effectively, it ensures that reported data accurately reflect provider performance by identifying and correcting errors, preventing fraud and overreporting, recovering costs, and enforcing penalties, thereby strengthening transparency and accountability [15,16]. Verification can take place at various levels: within health facilities to assess service quantity and quality, at the community level to validate reported services and gauge patient satisfaction, or at the management level to evaluate regulatory compliance. While verification approaches vary across settings, they should prioritize credibility, sustainability, and cost-effectiveness [15].

Some PBF programs incorporate counter-verification as an additional safeguard to reinforce the credibility of performance data. Counter-verification should ensure that verification processes are conducted rigorously, without data manipulation or bias. However, unlike verification, which has been widely studied and documented in the academic literature, counter-verification remains largely unexplored and underreported [17–19]. This study examines the counter-verification activities implemented by an independent external verification agency within the PBF program in Côte d’Ivoire. The findings contribute to broader efforts to integrate strategic health purchasing into health financing systems in Côte d’Ivoire and other sub-Saharan African countries.

### Context and main features of PBF in Côte d’Ivoire

Before adopting PBF as a national policy in 2019, Côte d’Ivoire piloted three PBF schemes. The first pilot, implemented by the Elizabeth Glaser Pediatric AIDS Foundation (EGPAF) from 2006 to 2009, targeted 32 public and private health facilities across 21 health districts. Its primary objective was to increase health service utilization and improve service delivery for HIV/AIDS care [20]. The second pilot, conducted by Abt Associates from 2009 to 2011 in a single district, similarly focused on enhancing HIV/AIDS-related indicators and retaining healthcare workers in underserved areas [20].

Building on insights from these initial pilots [20], the government launched a third pilot between 2016 and 2019, covering 21 of the country’s 113 health districts. This phase was co-financed by the national budget, the World Bank, and other donors, including the Global Fund, UNICEF, and USAID. In 2019, PBF was expanded nationwide to all 113 health districts and 33 health regions, with the World Bank as the primary financer, although the Ivorian government was also expected to contribute.

The institutional framework for PBF in Côte d’Ivoire was designed to ensure a clear separation of functions. The regulatory and coordination roles were assigned to central, regional, and district-level health administrators, as well as a National Steering Committee and a National Technical Unit for PBF, which operated under the Ministry of Health. Service provision was carred out by approximately 2,400 primary healthcare (PHC) facilities and 120 secondary hospitals, while tertiary hospitals and private healthcare providers were not included at the time of writing. The purchasing and payment functions were managed by the World Bank’s Health Projects Coordination Unit. Verification is a multi-layered process involving several actors. At the facility level, three national contracting and verification agencies (CVAs) conducted monthly checks on service quantity, while district and regional health management teams conducted quarterly assessments of service quality. Community verification, though irregular, was carried out by community-based organizations under performance contracts with the CVAs, which also provided training and oversight. This process serves several purposes: i) verifying the existence of patients or clients recorded in quantitative verification; ii) confirming that reported services were indeed provided and received; and iii) assessing patient satisfaction regarding respect, attentiveness, and service quality. Additionally, community verification incorporates beneficiary feedback to improve healthcare service quality and accessibility. Regulatory oversight was conducted quarterly, with regional health management teams (RHMTs) supervising district health management teams (DHMTs) and the National Technical Unit for PBF overseeing RHMTs. The National Technical Unit also evaluated CVAs’ performance. Since 2020, an international independent external verification agency, Ginger INTERNATIONAL (IEVA/GI), has been responsible for quarterly counter-verification activities.

The National Technical Unit for PBF manages a dedicated web portal (https://data.fbpcotedivoire.org/) designed to facilitate the efficient administration of PBF activities. This platform enables the entry of verified data; the monitoring and evaluation of key performance indicators; and the tracking of payments for all entities operating under a performance contract.

### Scope and methodology for counter-verification in Côte d’Ivoire

**Figure 1.**
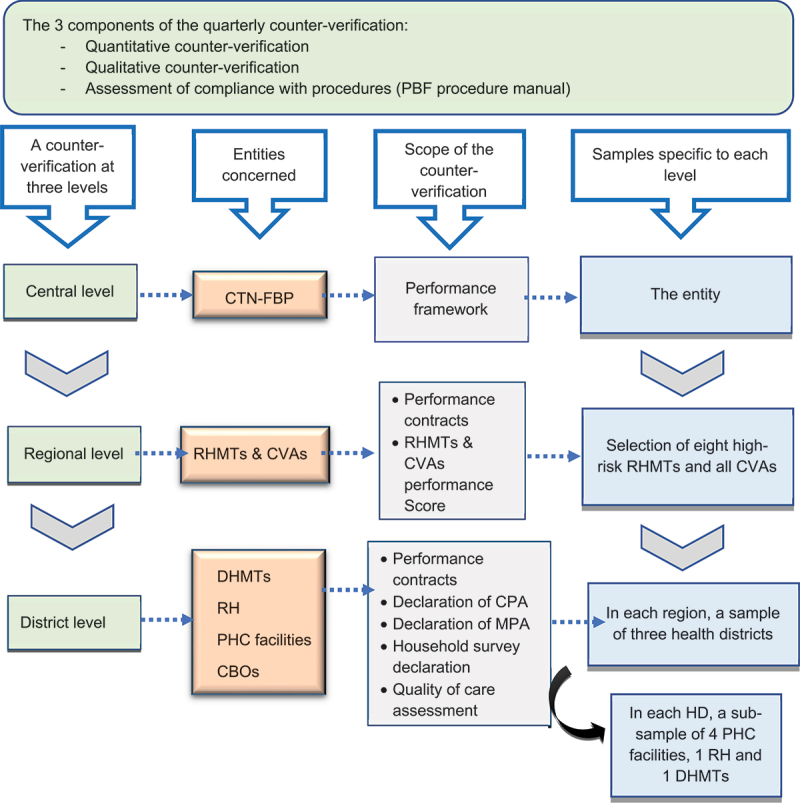
Entities counter-verified, their selection process, and the scope of verification by health system level.

Source: Technical offer – Ginger INTERNATIONAL based on the PBF Procedures Manual, June 2021 version

Counter-verification in Côte d’Ivoire covers all verification activities and follows a three-tiered sampling approach to select the PHC facilities. This approach combines purposive, systematic, and random sampling, considering factors such as perceived risk, the number of previous counter-verifications, and the total number of facilities within a health district. The process begins with the selection of regional health departments, followed by health districts and, finally, PHC facilities. Referral hospitals, including regional and district hospitals, as well as health management teams at the regional and district levels, are systematically included in the sample. The scope and methodology for these selections are detailed in Figure 1.

Once referral hospitals and PHC facilities are chosen, the indicators for counter-verification are randomly selected. Additionally, the IEVA/GI conducts two assessments that are not subject to prior verification: i) the evaluation of archiving and documentation management systems and ii) the assessment of compliance with financial management procedures. The latter includes administrative and financial audits of invoices, as well as the verification of adherence to financial and accounting procedures in the use of PBF resources.

## Methods

This study is based on documentary analysis and informal, rapid discussions with the IEVA/GI staff involved in drafting the documents. These discussions, typically conducted remotely via phone, Zoom, or WhatsApp, occurred, while the documents were being reviewed to ensure a thorough understanding. For the documentary analysis, we applied the READ approach proposed by Dalglish et al. [21], which involves a four-step process: i) readying materials, ii) extracting data, iii) analyzing data, and iv) distilling findings.

### Readying materials (READ)

The study utilized two types of documents provided by the IEVA/GI: (i) quarterly reports of counter-verification missions and (ii) Excel database files analyzing quantitative data on the services delivered during each mission. A total of 14 counter-verification missions were conducted between September 2020 and December 2023, covering the period from the fourth quarter of 2016 to the third quarter of 2023. This timeframe includes all activities that underwent counter-verification by the IEVA/GI, from their initiation to the most recent activities at the time of the study. The reports followed a standard structure, comprising an introduction to PBF in Côte d’Ivoire, a description of the mission’s objectives and tasks, an explanation of the methodology, findings, and a conclusion with recommendations.

### Extracting data (READ)

Relevant information from the reports was manually collected using a data extraction grid created in a Word document. The extraction focused on the IEVA/GI’s primary activities, including counter-verification of verified activities at health facilities, regulatory bodies, CVAs, and the community level, as well as assessment of the filing and archiving system and compliance with financial management procedures. Each counter-verification report addressing these areas was thoroughly read, and the pertinent sections were extracted and summarized.

### Analyzing data (READ)

We analyzed the data by referencing the benchmarks and guidelines outlined in the PBF management manuals, as reported in the counter-verification reports. The PBF Operational Manual permits a margin of error of ±10% to assess discrepancies between verified and counter-verified data. Any results outside this range are considered problematic and warrant further investigation to identify the underlying causes. The assessment results were also compared with the established best practices in document management, filing, and archiving, as well as sound financial management principles, with explanations provided for any observed shortcomings.

### Distilling findings (READ)

The findings are presented based on the data extraction items. First, we provide an overview of the discrepancies between the verification and counter-verification results, along with the primary reasons for these discrepancies. Second, we discuss the issues and challenges identified during the assessment of the archiving and documentation management system, as well as the financial management procedures, and examine the underlying causes.

### Positionality and reflexivity

Some of the coauthors were directly involved in implementing counter-verification activities, either through fieldwork (JPT, RYK, and SMM) or through commissioning (MPDS and XL) or funding (CKK) these activities. As insiders, there is a risk of overlooking certain aspects due to their familiarity. To address this, we contributed to the study design by highlighting key areas for investigation but did not participate directly in the data analysis. The analysis was conducted by JAK and KSBB, who were independently recruited for this purpose. The local context expertise of JPT, RYK, and SMM was crucial in interpreting and clarifying the results, enhancing the depth of understanding. This approach helped mitigate potential conflicts of interest, as JAK and KSBB were not involved in the implementation, verification, or counter-verification of PBF in Côte d’Ivoire, ensuring an objective analysis and interpretation of the data.

## Results

During the 14 counter-verification missions, the IEVA/GI conducted visits across all 33 health regions and 113 health districts of Côte d’Ivoire. Each region was visited between one and five times, while the districts were visited between one and three times. However, the counter-verifications for the CVAs and at the community level were not conducted the expected five and eight times, respectively, due to the absence of initial verifications. The documents did not provide any explanation for this omission. The following sections present the assessment results on: i) discrepancies between verified and counter-verified data, along with contributing factors; ii) the archiving and documentation management system; and iii) compliance with financial management procedures.

### Assessment of the discrepancies between the verified and counter-verified data revealed mixed results

Across all counter-verified entities, except for RHMTs and CVAs, the average discrepancy between verified and counter-verified data exceeded 10% in the 14 missions, indicating a significant level of variation. Discrepancies ranged from 6.1% for CVAs performance assessments to 22.5% for referral hospital quantity assessments, as detailed in Table 1. Supplementary file 1 provides graphical representations of the evolution of these discrepancies across missions in relation to the 10% threshold.

**Table 1.** Overview of the differences between the verified and counter-verified data.

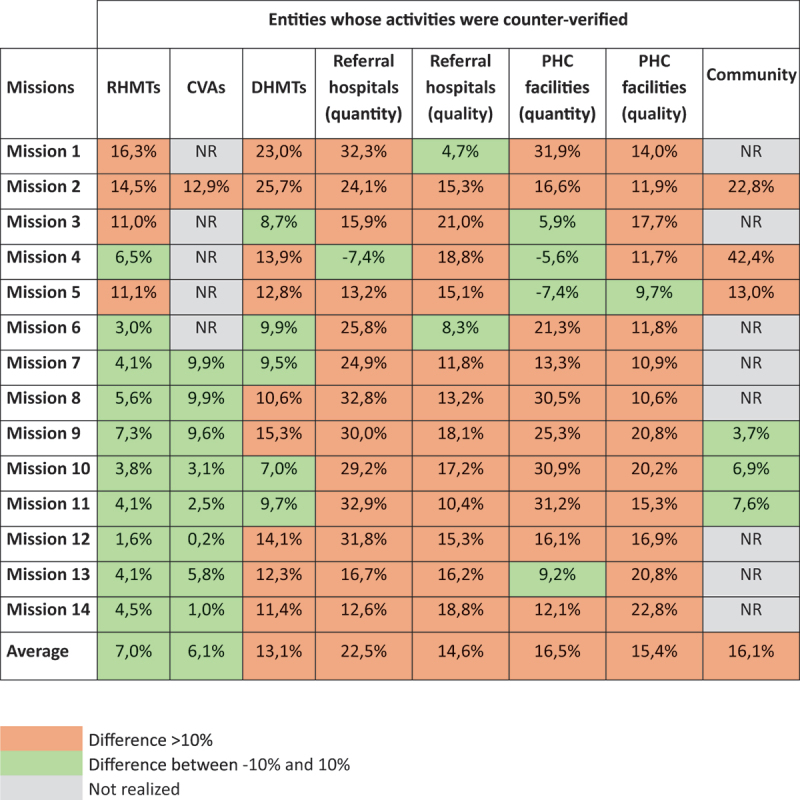

The last counter-verifications for RHMTs and CVAs at the community level showed discrepancies within the accepted range of ±10%, indicating a stable and reliable verification process over time. In contrast, referral hospitals and PHC facilities frequently exhibited significant discrepancies (>10%) in both quantity and quality assessment, with no clear trend toward improvement.

For quantity counter-verifications, referral hospitals had only one mission – the fourth – where discrepancies fell within the accepted range (−7,4%). The data revealed a downward trend from the first to the fourth missions (32.3% to −7.4%), followed by an overall increase from the fifth to the twelfth missions (13.2% to 31.8%), and a subsequent decline in the thirteenth and fourteenth missions (16.7% and 12.6%, respectively). A similar pattern was observed in PHC facilities, with fluctuations between the first and fifth missions (31.9% to −7.4%), the sixth and eleventh missions (21.3% to 31.2%), and the twelfth and fourteenth missions (16.1%, 9.2%, and 12.1%, respectively). In total, only four of the 14 missions (third, fourth, fifth, and thirteenth) had discrepancies within the accepted range.

For quality counter-verifications, referral hospitals showed discrepancies within the accepted range in only the first and sixth missions (4.7% and 8.3%, respectively). In other missions, discrepancies ranged from 10.4% (eleventh mission) to 21% (third mission). For PHC facilities, only the fifth mission had a discrepancy within the accepted range (9.7%). While discrepancies remained slightly above the 10% threshold between the first and eighth missions, they increased significantly from the ninth mission onward. For DHMTs, results fluctuated around the 10% threshold.

### Many challenges undermined data accuracy

The observed discrepancies can be attributed to several factors, primarily related to weaknesses in data recording and reporting in registers and other supporting documents used for counter-verifications. In some cases, key information was either missing from archives or inaccurately recorded, failing to meet the required standards for validating performance indicators.

One major challenge was the failure of teams to conduct self-assessments before the audits. Without proactive internal reviews, potential discrepancies and weaknesses remained unaddressed, reducing the opportunity to correct errors before external counter-verifications took place. Additionally, the inadequate implementation of previous recommendations further exacerbated data inconsistencies. Many issues identified in earlier verifications persisted over time, reflecting a lack of follow-up mechanisms and insufficient resources and training to enforce corrective actions. Another critical factor was the poor quality of reporting deliverables. Many reports did not adhere to established standards, often lacking essential elements such as signatures, attendance lists, or clear recommendations.

### Weaknesses were identified in the archiving and documentation management system, contributing to the discrepancies observed

Counter-verification missions revealed significant shortcomings in the physical and electronic archiving systems, primarily due to a lack of coordination and proper management. Documents were not systematically filed in chronological order, making it difficult to retrieve specific records for counter-verification. This issue persisted through all 14 missions, highlighting a structural problem in document management.

A major challenge was the inadequate storage capacity for hard-copy documents. There were no dedicated spaces for storing large volumes of records, and available shelving and storage boxes were insufficient to accommodate the growing number of documents. As a result, hard copies were often found in a poor condition, scattered on the floor or in corridors, increasing the risk of loss or misplacement.

While some premises were equipped with computers, printers, scanners, and internet access to facilitate electronic archiving, the lack of a standardized system for digital document management remained a critical gap. Documents were stored haphazardly on local computers or in email accounts without a structured organization, significantly limiting accessibility and retrieval efficiency.

Health facilities lacked a dedicated, qualified archivist to manage documents and archives, leading to the absence of a centralized and structured archiving system. Instead, each staff member was responsible for managing the documents they produced or oversaw, resulting in a fragmented and inconsistent approach to document storage. This posed a risk, as the absence of a specific individual could render critical documents inaccessible, disrupting counter-verification processes and overall data management.

However, several solutions could address these challenges and improve document accessibility. Implementing shared physical storage solutions, such as external hard drives, could help consolidate records. Additionally, interconnecting computers within facilities or establishing cloud-based shared files would provide centralized access to documents, ensuring continuity and efficiency even in the absence of the specific personnel.

### Complying with administrative and financial procedures sometimes proved challenging

The administrative and financial control of invoices primarily involved comparing discrepancies between amounts reported in health facilities’ data declaration and validation forms and those generated from data entered in the PBF portal. This control was conducted in eight of the 14 missions. The sixth, seventh, tenth, eleventh, twelfth, and fourteenth missions were unable to perform these controls due to portal maintenance or upgrades, delays in data entry by the CVAs, or pending approval of invoices by the national authority. The observed discrepancies were minor, within ±10% of the validated amount, and were primarily attributed to data entry errors.

However, an analysis of the health facility data declaration and validation forms revealed several issues that sometimes compromised their validity. These included the use of pens with different inks on the same invoice, suggesting potential forgery; the use of pencils, which allow for easy erasure and modification; the absence of a validation report for data submitted for payment; missing facility or responsible person seals, as well as forms that were not signed by the CVAs, as approval.

Health facilities are required to use PBF resources in compliance with specific procedures and regulations. The assessment evaluated six key areas: i) the availability of resource planning and management documents and tools, including the performance contract and the participatory budgeted business plan; ii) procurement of goods and services for operations; iii) staff bonus payments; iv) management of petty cash accounts; v) availability and archiving of supporting documents; and vi) availability of financial monitoring and reporting documents.

The evaluation results were generally satisfactory or good for the first three areas but revealed significant weaknesses in the last three. Health facility managers often refrain from using petty cash funds due to cumbersome administrative procedures; when they do, they frequently fail to comply with the required processes. Furthermore, key supporting documents for the procurement of goods and services and the payment of staff bonuses – such as requests for quotations, selection reports, purchase orders, delivery notes, invoices, and validated payment orders – were often missing, incomplete, or improperly archived. Similar issues were noted for financial monitoring and reporting documents, including monthly bank statements, bank reconciliations, and financial reports on PBF fund utilization.

## Discussion

### Counter-verification as a credibility measure: challenges in data quality and system reliability

Counter-verification serves as a critical tool for assessing and enhancing the credibility of the verification process. Taking counter-verification as the gold standard, findings from Côte d’Ivoire indicate significant weaknesses in the verification process, particularly in healthcare facilities and DHMTs. These shortcomings were evident in both the quantity and quality assessments of services, largely due to persistent issues with data quality and validity. Specifically, deficiencies in data recording, storage, and archiving – both before and after verification – undermine the reliability of the process.

The findings of this study suggest that the PBF has not fully achieved one of its primary objectives: improving data quality and enhancing the reliability of Health Management Information System (HMIS) data [21]. Data quality challenges have continued to persist across multiple counter-verification missions, despite the particular emphasis often placed on the quality of the tools and processes supporting the HMIS. In many countries implementing PBF, criteria such as record keeping, data archiving, and data reporting are commonly included as performance indicators tied to payment [19,23]. This highlights that PBF is not a universal solution that automatically delivers the intended results [24,25], but rather serves as a catalyst for change [6]. To maximize its effectiveness in strengthening HMIS, it is essential to conduct a thorough analysis of the underlying issues and address deficiencies identified during the verification phase promptly, rather than allowing them to persist until the counter-verification phase. This proactive approach would improve the overall impact of PBF on health information systems.

To maximize the effectiveness of PBF, deliberate investment and sustained efforts are required. Key priorities include ensuring a clear understanding of performance indicators and their validation criteria, establishing a robust data collection system with internal verification mechanisms to prevent inconsistencies or missing data, developing a reliable data storage and archiving system, empowering stakeholders to improve compliance, fostering behavioral change, and reinforcing accountability mechanisms. These measures are essential for strengthening the credibility of verification and counter-verification processes and ultimately enhancing health system performance.

### Counter-verification outcomes: variability across system levels and the role of community verification

Counter-verification results for RHMTs and CVAs were generally more favorable, with discrepancies remaining below 10% from the sixth and seventh missions onward. In contrast, results for DHMTs were more inconsistent, with discrepancies fluctuating above and below the 10% threshold, while the latter was mostly exceeded for referral hospitals and PHC facilities. The stronger performance of RHMTs and CVAs may be attributed to their smaller numbers and relatively stable staff, which facilitate closer supervision and more effective implementation of recommendations made during verification missions. The situation is more complex at the level of DHMTs, PHC facilities – which are more numerous and geographically peripheral – and their referral hospitals, given the persistent challenges related to governance, human resources, infrastructure, equipment, supplies, and financing [26–30]. These factors may create disincentives or lead to the selection of indicators that are less responsive to improvement. While PBF is intended to help address these issues [6], our findings indicate that achieving sustainable change requires a combination of concerted efforts and other structural reforms.

Community verification and counter-verification were conducted six times, though not on a regular basis. However, the last three counter-verifications showed discrepancies of less than 10%, suggesting a gradual improvement over time. Beyond identifying fictitious patients and incorrect diagnoses or prescriptions, community verification within PBF provides an additional benefit: it amplifies patients’ voices by capturing their perceptions, levels of satisfaction, and concerns regarding the care they receive [22]. This makes it a powerful tool for strengthening healthcare providers’ accountability to the public, although its effectiveness is not always guaranteed [31].

Despite its potential, community verification often faces financial, logistical, and operational constraints [18,32,33], which likely explain its irregular implementation in Côte d’Ivoire. Similar challenges have been observed in other countries [18,33], highlighting the need for greater investment and strategic planning to enhance its sustainability and impact.

### Balancing cost and value: the role of counter-verification in PBF

PBF has been criticized for its costly verification process, raising concerns about its overall cost-effectiveness and value for money [1,18,34–36]. As a result, many countries are increasingly encouraged – or are voluntarily shifting – toward risk-based verification approaches that prioritize efficiency [17,37]. In this context, adding a counter-verification component may seem contradictory, particularly in Côte d’Ivoire, where the discrepancies observed between verified and counter-verified data suggest that the financial savings from preventing fraud, over-reporting, or gaming are minimal and may not justify the resources allocated to counter-verification.

However, the benefits of well-executed counter-verifications extend beyond financial savings. It serves as a deterrent against data manipulation, enhances transparency, and strengthens the credibility of the reported data. These factors, in turn, foster mutual trust among stakeholders in the healthcare system and promote evidence-informed decision-making [38,39].

To optimize costs while maintaining its added value, counter-verification can be strategically targeted and risk-based, like verification itself. By focusing on high-risk areas and using counter-verification findings to identify key action points for capacity building and system improvement, countries can ensure that the process remains both cost-effective and impactful. Additionally, data digitization and artificial intelligence can be leveraged to perform automated verifications through the development of contextual algorithms that are regularly updated and refined. It is also essential to integrate verification and counter-verification processes into routine activities, minimizing the additional workload for health facility staff and ensuring financial sustainability for the health system.

### Study limitations

Despite its valuable insights, this study has several limitations. It relies primarily on counter-verification mission reports, which may omit undocumented verification challenges or contextual factors affecting data discrepancies. The accuracy of findings depends on the completeness and objectivity of these reports, making them susceptible to biases, omissions, or inconsistencies. Additionally, the study lacks direct interviews, field observations, or independent data verification, which could have provided a more comprehensive understanding of discrepancies and verification challenges. To mitigate these limitations, future research could complement document review with qualitative interviews, field validation, and comparative analyses across different PBF programs.

## Conclusions

Counter-verification plays a critical role in strengthening the credibility of the verification process, a cornerstone of any PBF program. It serves as a safeguard against both intentional misconduct and unintentional errors, enhancing transparency and accountability in healthcare service delivery. However, this study identified persistent discrepancies exceeding 10% between verified and counter-verified data, highlighting structural and operational challenges that undermine the effectiveness of PBF. Addressing these issues is essential to maximizing the program’s impact on health system performance. To enhance the effectiveness and sustainability of counter-verification, several key elements should be prioritized. These include systematically integrating counter-verification into PBF design, adopting a risk-based and targeted approach, strengthening data management systems, investing in capacity building and stakeholder engagement, and ensuring continuous evaluation and adaptation to improve efficiency and responsiveness.

## Supplementary Material

Supplementary file 1.docx

## Data Availability

The datasets used and analyzed in this study are available from the IEVA/GI and can be obtained upon reasonable request.
