# Supplementary material for: Counter-verification in performance-based financing: key insights from the Côte d’Ivoire experience
Source: Glob Health Action. 2025 Dec 18;18(1):2483072. doi: 10.1080/16549716.2025.2483072 (PMC12716480; doi:10.1080/16549716.2025.2483072)
Supplement: Supplementary file 1.docx [file ZGHA_A_2483072_SM0694.docx]

**Supplementary file 1**

**Figure S1. Evolution of the average discrepancies in the scores between the verification and the counter-verification of the performance of the regional health management teams (RHMTs) for the 14 missions**

**Figure S2. Evolution of the average discrepancies in the scores between the verification and the counter-verification of the contracting and verification agencies (CVAs) for the 2^nd^ and 7^th^ to 14^th^ missions**

**Figure S3. Evolution of the average discrepancies in the scores between the verification and the counter-verification of the performance of the district health management teams (DHMTs) for the 14 missions**

**Figure S4. Evolution of the average discrepancies in the scores between the verification and the counter-verification of the quantity check of the referral hospitals for the 14 missions**

**Figure S5. Evolution of the average discrepancies in the scores between the verification and the counter-verification of the quality check of the referral hospitals for the 14 missions**

**Figure S6. Evolution of the average discrepancies in the scores between the verification and the counter-verification of the quantity check of the primary health care (PHC) facilities for the 14 missions**

**Figure S7. Evolution of the average discrepancies in the scores between the verification and the counter-verification of the quality check of the PHC facilities for the 14 missions**

**Figure S8. Evolution of the average discrepancies in the scores between the verification and the counter-verification of the community survey for the 2^nd^, 4^th^, 5^th^, 9^th^, 10^th^, and 11^th^ missions**
